# Multifocal skeletal tuberculosis in an immunocompetent patient: a case report

**DOI:** 10.1186/s12879-015-0985-x

**Published:** 2015-06-20

**Authors:** Paulo Sérgio Ramos de Araujo, Heloisa Ramos Lacerda de Melo, Fábio Lopes de Melo, Zulma Medeiros, Maria Amélia Maciel, Renata Florêncio, Eduardo Brandão

**Affiliations:** Service of Infectious and Parasitic Diseases, Department of Tropical Medicine, Hospital de Clinicas, Federal University of Pernambuco, Cidade Universitária, Av. Prof. Moraes Rego, 1235, Recife, PE CEP: 50.670-901 Brazil; Aggeu Magalhães Research Center, FIOCRUZ, Cidade Universitária, Av. Prof. Moraes Rego, 1235, Recife, PE CEP: 50.670-901 Brazil

**Keywords:** Multifocal, Skeletal, Tuberculosis, Immunocompetent, Atypical presentation

## Abstract

**Background:**

The occurrence of multifocal skeletal involvement in immunocompetent patients is rare, even in countries where tuberculosis is endemic. Multifocal skeletal lesions may occur as a result of hematogenous dissemination from another primary focus such as cervical lymph nodes, lungs, tonsils or gastrointestinal tract.

**Case presentation:**

We present a 59 year-old man with a history of intermittent and disabling pain in his left knee for 2 years. The patient in this case presented with lung infection with bilateral skeletal dissemination in the knees and femurs. Immunological examination for the HIV was negative.

**Conclusions:**

Diagnosis of this condition is not always easy because of the disease’s insidious character, and it can be confused with other diseases such as osteoarthritis, especially in middle-aged individuals.

## Background

Skeletal tuberculosis represents less than 2 % of all tuberculosis cases and may affect one or more joints. Joints that support greater weight loads, such as the knees and hips, are the most affected in cases of skeletal tuberculosis [1]. The involvement of multiple joints is rare in immunocompetent patients [2, 3]. The symptoms are nonspecific, and the disease often presents with an indolent clinical course, usually leading to a delayed diagnosis as well as bone and joint destruction. A substantial number of patients report symptoms including pain and discomfort for over a year with reports of unsuccessful treatments for osteoarthritis [4, 5].

The patient in this case presented with lung infection with bilateral skeletal dissemination in the knees and femurs. In such cases, the diagnosis must be made the basis of the isolation of *Mycobacterium tuberculosis* in Lowenstein–Jensen culture medium and susceptibility testing using bone and/or synovial biopsy. Molecular methods such as real-time polymerase chain reaction (PCR) are much more sensitive and specific.

Treatment should preferably be drug-based with a combination of 4 anti-tuberculosis drugs for 6 months. This period may be extended in cases of poor response. As these drugs do not penetrate the bone or fibrous tissue deeply, some guidelines recommend extended treatment up to 12–18 months; treatment durations as long as 4 years have been reported [6–9]. Here, we present a case of multifocal skeletal tuberculosis affecting the knees in an immunocompetent individual, which progressed with a slow therapeutic response to a fixed-dose combination regimen of anti-tuberculosis drugs.

## Case presentation

**Fig. 1:**
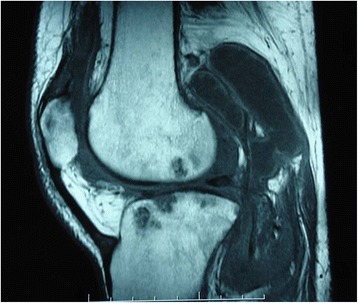
Magnetic resonance imaging showed signs of osteomyelitis in the right femur and tibia

A 59 year-old man presented with a history of intermittent and disabling pain in his left knee for 2 years. There were no other complaints such as fever, weight loss, or coughing. He had undergone arthroscopy a year earlier to treat osteoarthritis. A second arthroscopy was performed with synovial tissue biopsy in July 2010; microbiological analysis revealed the presence of *M. tuberculosis* and acid-fast bacilli (AFB). Antibiogram showed a normal sensitivity pattern. Histopathological assessment revealed granuloma with caseous necrosis, suggesting tuberculosis. Chest radiography revealed bilateral diffuse pulmonary infiltrates with a micronodular pattern. Immunological examination for the human immunodeficiency virus (HIV) was negative. The patient began receiving treatment with anti-tuberculosis drugs as part of a regimen, including rifampicin, isoniazid, pyrazinamide, and ethambutol. Pain symptoms initially improved. However, 2 months after starting the treatment, he started experiencing pain and swelling, and developed a fistula in the contralateral knee. Magnetic resonance imaging (MRI) showed signs of osteomyelitis in the right femur and tibia (Fig. 1), and surgical drainage with bone biopsy suggested tuberculosis. However, there was no microbiological growth in mycobacteria-selective culture medium. After 6 months of treatment, the patient presented with a fistula in his left knee, which was positive for AFB. *M. tuberculosis* was isolated from a selective medium in a reference laboratory. However, sensitivity tests could not be performed. Once again, immunological assessment and real-time PCR results were negative for HIV. Immunoglobulin values were normal. His therapeutic regimen was subsequently extended. In the eighth month of treatment, the fistula was still observed in his right knee, and the collected material revealed the presence of AFB. MRI revealed the presence of periarticular collection. One year after the initiation of isoniazid/rifampicin treatment, there were no fistulas and findings of MRI suggested improvement. In August 2012, after 24 months of isoniazid/rifampicin treatment, the patient returned to the outpatient clinic presenting with a new fistula in his right knee. MRI revealed periarticular and intraosseous collection in the left femur as well as a large collection in the right calf. Surgical drainage of a large volume of purulent material was performed, and the contents were sent to 3 laboratories; *M. tuberculosis* was not isolated in any of the laboratories, but the results of tests conducted in 2 laboratories indicated that the material was positive for AFB and *M. tuberculosis* was detected by real-time PCR. By February 2013, the patient had been treated with isoniazid/rifampicin for 30 months and he began to experience clinical improvement with the disappearance of the fistulas. The final MRI scan showed improvement of osteomyelitis signs. The treatment was discontinued in January 2013, and the patient is currently in clinical follow-up.

## Conclusion

Multifocal skeletal tuberculosis, one of the various forms of extrapulmonary tuberculosis, is uncommon. Diagnosis of this condition is not always easy because of the disease’s insidious character, and it can be confused with other diseases such as osteoarthritis, especially in middle-aged individuals. Delayed diagnosis may result in the disease spreading to adjacent bone structures, leading to difficulty in clinical management.

### Consent

Written informed consent was obtained from the patient for publication of this Case report and any accompanying images. A copy of the written consent is available for review by the Editor of this journal.
